# UHPLC-MS-Based Metabolomics Reveal the Potential Mechanism of *Armillaria mellea* Acid Polysaccharide in and Its Effects on Cyclophosphamide-Induced Immunosuppressed Mice

**DOI:** 10.3390/molecules28247944

**Published:** 2023-12-05

**Authors:** Ying Li, Qingqing Li, Huazhou Niu, Hui Li, Lili Jiao, Wei Wu

**Affiliations:** Jilin Ginseng Academy, Changchun University of Chinese Medicine, Changchun 130117, China; ying060111@163.com (Y.L.); liqqsunny@126.com (Q.L.); niuhuazhou95@163.com (H.N.); lihuiterrisa@163.com (H.L.); jiaoaj@hotmail.com (L.J.)

**Keywords:** *Armillaria mellea*, acid polysaccharide, UHPLC-MS, metabolomics

## Abstract

*Armillaria mellea* (Vahl) P. Kumm is commonly used for food and pharmaceutical supplements due to its immune regulatory function, and polysaccharides are one of its main components. The aim of this research is to study the immunological activity of the purified acidic polysaccharide fraction, namely, AMPA, isolated from *Armillaria mellea* crude polysaccharide (AMP). In this study, a combination of the immune activity of mouse macrophages in vitro and serum metabonomics in vivo was used to comprehensively explore the cell viability and metabolic changes in immune-deficient mice in the AMPA intervention, with the aim of elucidating the potential mechanisms of AMPA in the treatment of immunodeficiency. The in vitro experiments revealed that, compared with LPS-induced RAW264.7, the AMPA treatment elevated the levels of the cellular immune factors IL-2, IL-6, IgM, IgA, TNF-α, and IFN-γ; promoted the expression of immune proteins; and activated the TLR4/MyD88/NF-κB signaling pathway to produce immunological responses. The protein expression was also demonstrated in the spleen of the cyclophosphamide immunosuppressive model in vivo. The UHPLC-MS-based metabolomic analysis revealed that AMPA significantly modulated six endogenous metabolites in mice, with the associated metabolic pathways of AMPA for treating immunodeficiency selected as potential therapeutic biomarkers. The results demonstrate that phosphorylated acetyl CoA, glycolysis, and the TCA cycle were mainly activated to enhance immune factor expression and provide immune protection to the body. These experimental results are important for the development and application of AMPA as a valuable health food or drug that enhances immunity.

## 1. Introduction

*Armillaria mellea* (Vahl) P. Kumm is a common edible fungus containing a variety of nutrients. Polysaccharides of Armillaria have been studied for their immunomodulatory and tumor-suppressive effects in modern pharmacognosy and pharmacology. Polysaccharides are the main component of Armillaria usually found in its fruiting bodies and their hyphae [1,2,3]. The polysaccharides were extracted from the fruiting bodies of Armillaria and evaluated in vitro to assess their immunomodulatory activity using jack bean protein A or lipopolysaccharide [2,4]. Therefore, exploring the biological mechanisms of immunoregulation to obtain safer and more effective functional foods and pharmaceutical supplements is necessary in contemporary research.

In in vitro immunoactivity studies of plant polysaccharides, RAW264.7 is used to examine the mechanism of the modulatory impacts on immune function, whereas immunodeficient mice (induced by cyclophosphamide (CTX)) are usually studied as experimental animal models. In vivo CTX destroys mouse macrophages and suppresses the expression of immune factors, mimicking the immunodeficiency state [5,6]. It has been stated that mouse macrophages (RAW 264.7) are an important target cells for immunomodulatory activities. Additionally, in vitro, plant and fungus polysaccharides activate RAW 264.7 cell proliferation and are usually utilized to establish immunodeficiency models to simulate immunodeficiency resistance in patients with immunoregulation. For example, *Undaria pinnatifida* polysaccharides can activate the TLR 4 receptors engaged in NF-κB p65 signaling and drive the immunomodulatory phenotype [7]; the Astragalus polysaccharide promotes the NF-κB protein to activate the p65/MAPK signaling pathway [8,9]. Plant polysaccharides activate the TLR4/MyD88/NF-κB signaling pathway mostly through stimulating an immune response by inducing macrophages. It has also been observed that TLR4 can interact with MyD88, thereby leading to the activation of NF-κB, which is indispensable for the production of cytokines, such as IL-2, IgM, and TNF-α [10,11]. Accumulating evidence has emphasized the role of metabolites in the development of immunoregulatory. Metabolomics has become a powerful tool for screening the potential biomarkers and molecular mechanisms of traditional Chinese medicine for treating diseases [12,13,14,15]. It also has been successfully applied to the study of plant polysaccharide protection mechanisms on the immune suppression induced by CTX. Polysaccharide metabolites can activate acetyl CoA, glycolysis, and the TCA cycle and regulate the expression of immune factors. There is a clear correlation between immune cells, immune factors, and sugar metabolites, which play a wide range of physiological and pathological roles in immune responses [16,17]. This method provides a comprehensive qualitative analysis of metabolites in vivo, providing vital information on chemical biomarkers, and could be used for determining taxonomy and quality control. Accordingly, metabolomics could better demonstrate the key metabolites, pathways, and main regulatory processes of immunocompromised mice [18].

In the present study, cell model activity screening combined with metabolomics is used to explore the active components of AMPA isolated from Armillaria fruiting bodies. The potential secretion of immune-enhancing factors and activation-related signaling pathways of the macrophage RAW264.7 are investigated in vitro and verified through experiments using the immunosuppressive CTX mouse model. An analysis based on UHPLC-MS is used to screen the differential metabolites in mouse serum after 30 days of intervention. Combined with the enrichment analysis of the KEGG metabolic pathway, the related metabolic pathways of AMPA for treating immune deficiency and potential therapeutic biomarkers are explored.

## 2. Results

### 2.1. Effect of AMPA on RAW264.7 Cell Proliferation

To determine whether AMPA is cytotoxic to RAW264.7 cells, CCK-8 and cell phagocytosis experiments were performed to screen concentrations. The formula for the cell proliferation rate is as follows:

CCK-8 rate% = [(ODs − ODb)/(ODc − ODb)] × 100%(1)

In the formula, ODs is the absorbance of the treated group (including cells, medium, CCK-8 solution, and drug solution), ODc is the absorbance of the normal group (including cells, medium, CCK-8 solution, and without the drug), and ODb is the absorbance of the blank wells (including medium, CCK-8 solution, and without the cellular drug).

**Figure 1:**
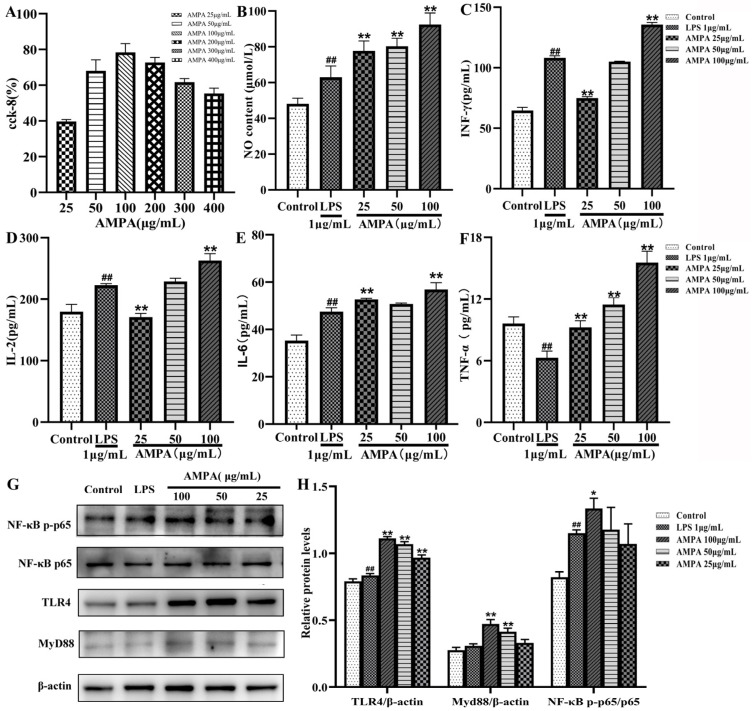
Effects of AMPA on CCK-8, NO, INF-γ, IL-2, IL-6, and TNF-α. (**A**) CCK-8, (**B**) NO, (**C**) INF-γ, (**D**) IL-2, (**E**) IL-6, (**F**) TNF-α, (**G**) representative Western blot diagram of RAW264.7, and (**H**) protein quantification of TLR4/β-actin, MyD88/β-actin, and NF-κB p-p65/p65. ## *p* < 0.01 compared with the control group. * *p* < 0.05 and ** *p* < 0.01 compared with the LPS treatment.

As shown in Figure 1A, the cell proliferation rate under the conditions of 200 μg/mL and 400 μg/mL of AMPA decreased significantly (*p* < 0.05). AMPA concentration values of 25.0 μg/mL, 50 μg/mL, and 100 μg/mL did not affect the normal proliferation of RAW264.7 cells. Based on these results, for the following experimental study of AMPA, the AMPA concentrations of 25.0 μg/mL, 50 μg/mL, and 100 μg/mL were selected.

### 2.2. Effects of AMPA on the Production of NO in RAW264.7 Cells

NO is a vital active substance related to immunomodulating responses to macrophage cells [19,20]. RAW264.7 cells were exposed to different concentrations of AMPA with NO concentrations, as shown in Figure 1B. The amount of NO released significantly increased in RAW264.7 cells, and NO production was significantly induced by the AMPA treatment compared with LPS induction in the cells. Considering the above results, the treatment with AMPA could remarkably increase NO secretion.

### 2.3. AMPA Promotes Cytokine Secretion by RAW264.7 Cells

The release of cytokines (INF-γ, TNF-α, IL-2, and IL-6) in AMPA-treated macrophage RAW264.7 cells was further evaluated using ELISA. The immune response associated with cytokine levels is a multistep process. These cytokines are all important cytokines that directly affect cellular immunity and humoral immunity. The effect of AMPA on humoral immunity was studied. As shown in Figure 1C–F, the levels significantly increased in the AMPA group compared with the LPS group, indicating that AMPA could increase the secretion of cytokines and induce cellular immune effects.

### 2.4. AMPA Activates the TLR4/MyD88/NF-κB Signaling Pathway in RAW264.7 Cells

The activation of the TLR4/MyD88/NF-κB signaling pathway mostly occurs during an immune response; it has been observed that TLR4 can interact with MyD88, thereby leading to the activation of NF-κB [21]. Hence, we investigated the proteins involved in the TLR4-mediated MAPK/NF-κB signaling pathway (Figure 1G,H). Compared with the control group, 200 μg/mL of AMPA significantly elevated the levels of TLR4 and MyD88. Moreover, AMPA notably promoted the phosphorylation of NF-κB p-p65/p65 in a dose-dependent manner. The treatment with 200 μg/mL of AMPA induced the expression of p-p65 by 1.62-fold in comparison to the control group. These results suggest that the AMPA induces macrophage activation and cytokine secretion via TLR4 and can interact with MyD88, thereby stimulating the activation of the NF-κB signaling pathway.

### 2.5. AMPA Improves the Physiological and Biochemical Parameters of CTX-Treated Mice

**Figure 2:**
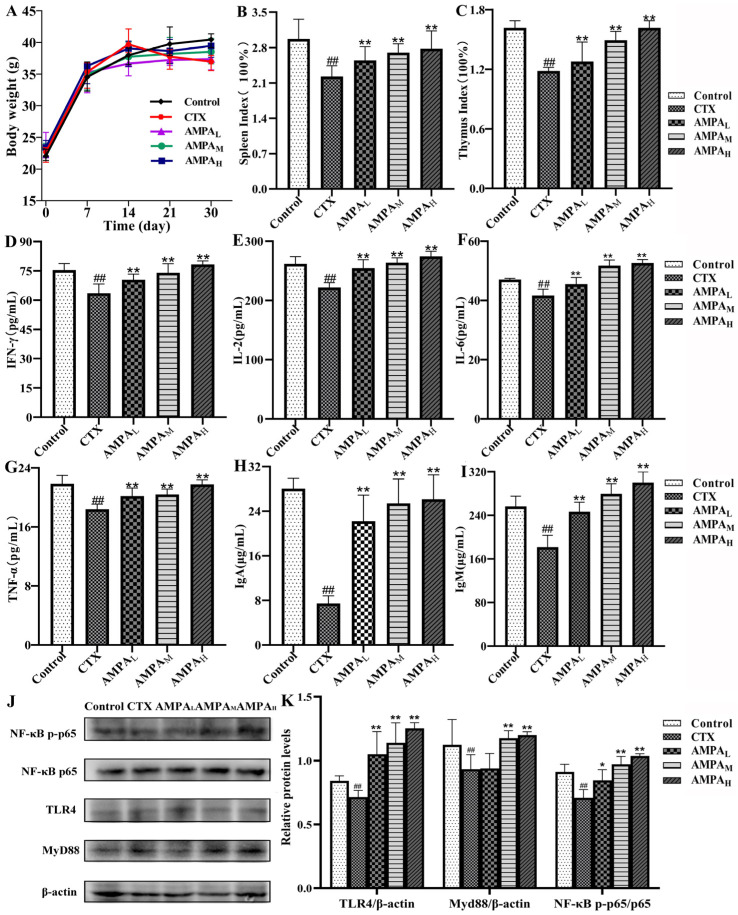
AMPA body weights (**A**), spleen index (**B**), and thymus index (**C**). Biochemical parameters of mice: (**D**) INF-γ, (**E**) IL-2, (**F**) IL-6, (**G**) TNF-α, (**H**) IgA, and (**I**) IgM. (**J**) Representative Western blot diagram of the spleen and (**K**) protein quantification of TLR4/β-actin, MyD88/β-actin, and NF-κB p-p65/p65. ## *p* < 0.01 compared with the control group. * *p* < 0.05 and ** *p* < 0.01 compared with the CTX treatment.

In order to test the immunostimulatory effect of AMPA on the immunosuppressed mouse model, mice were intraperitoneally injected with CTX (80 mg/kg·d) for 3 consecutive days after 24 days of administration of AMPA (50, 100, and 200 mg/kg·d). The weight results show that, compared with the blank group, the weights of the CTX group decreased significantly, while the weights of the AMPA group remained unchanged; the weights tended to increase after 7 days of continuous administration (Figure 2A). After 30 days of administration, the immune organs were weighed after dissection. The results show that the CTX injection significantly reduced the organ index of the spleen and thymus immune organs in the mice (*p* < 0.01). However, after the oral administration of AMPA, the organ index tended to return to the same level as that of the blank group, and those in the AMPA experimental group were reversed in a dose-dependent manner (Figure 2B,C).

ELISA was used to measure the quantities of immunoglobulins and immunological components in the blood of mice. Compared with the blank group, the CTX model group significantly reduced the concentrations of IL-2, IL-6, IgM, IgA, and TNF-α and IFN-γ immune factors (*p* < 0.01). The AMPA group exhibited a significant secretion of immune factors compared with the model group (*p* < 0.05 or *p* < 0.01). The above results show that AMPA could promote the expression of immune factors and activate an immune response. The results of serum biochemical marker results demonstrate that the oral administration of 200 mg/kg·d AMPA could effectively protect against immune damage (Figure 2D–I).

### 2.6. AMPA Activates the TLR4/MyD88/NF-κB Signal Pathway in CTX-Treated Mice

In the present study, the protein levels of MyD88/β-actin and TLR4/β-actin in the spleens of the CTX mice group remarkably decreased compared with the control mice, and the protein levels of NF-κB p-p65/NF-κB p65 were significantly decreased (*p* < 0.05; Figure 2J,K). In comparison with the model group, the AMPA intervention enhanced the protein expression levels of MyD88, TLR4, and NF-κB p-p65. The results show that AMPA promoted TLR4, MyD88, and NF-κB p-p65 protein expression in the spleen of immunodeficient mice, thus activating the TLR4/MyD88/NF-κB signaling pathway to achieve immune regulation, an effect that was dose-dependent.

### 2.7. UHPLC-MS-Based Serum Metabolomics Analysis

**Figure 3:**
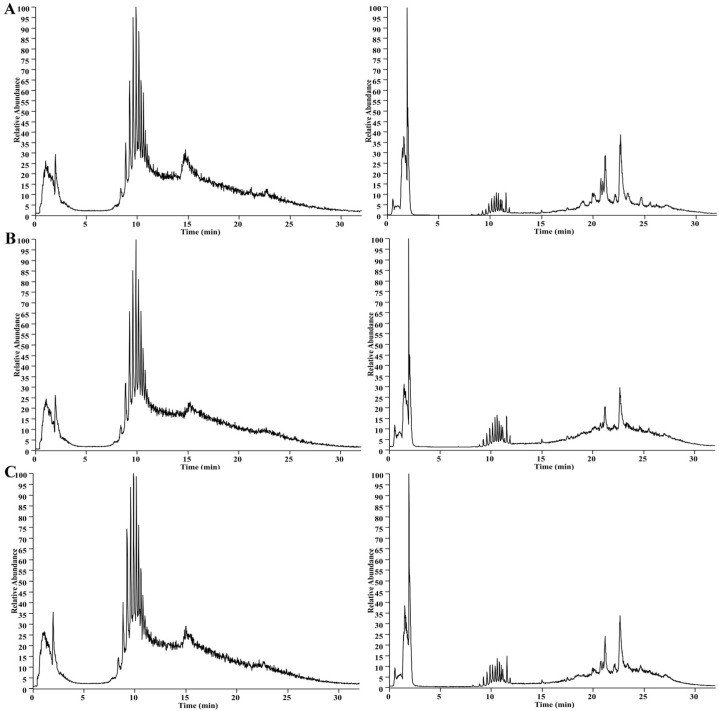
The representative UHPLC–MS TIC of the serum from the control group (**A**), CTX group (**B**), and AMPA group (**C**). The TIC on the left side shows the positive mode and on the right side shows the negative mode.

**Figure 4:**
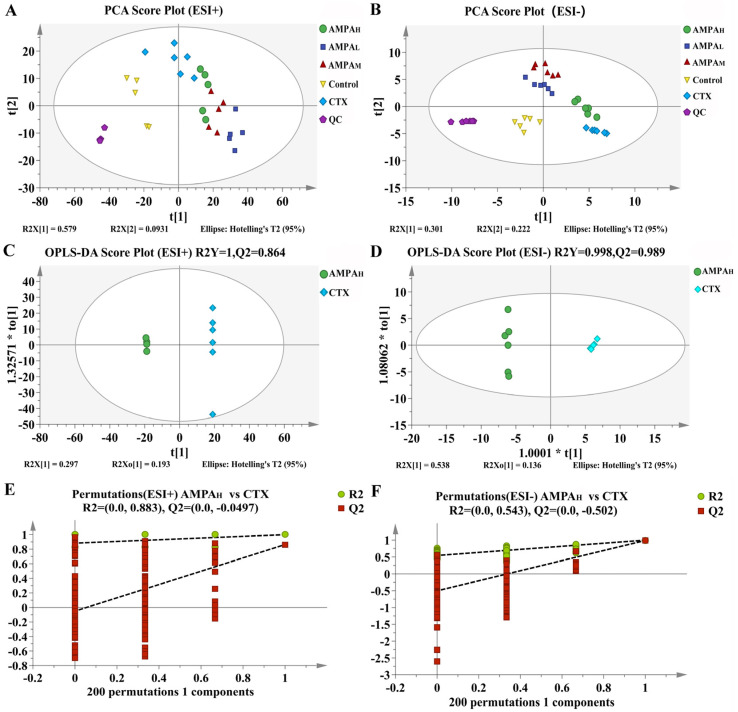
(**A**,**B**) PCA score plots in the positive and negative modes of the 5 comparison groups. (**C**,**D**) OPLS-DA score plots of the AMPA_H_ vs. CTX groups. (**E**,**F**) Permutation plot for 2 groups using the 200-response reciprocity test in the positive and negative modes for the AMPA_H_ vs. CTX groups.

The UHPLC-MS method was set up for analysis. Mice serum samples were analyzed via UHPLC-MS in the positive and negative modes; the collected TIC data are shown in Figure 3. To ensure the stability and reliability of the system and data, quality control (QC) samples were tested during the analysis and, then, PCA modeling was performed for all sample datasets. The QC samples showed a good aggregation status in the PCA score (Figure 4A,B), indicating stable experimental conditions from the first sample to the last sample; appropriate chromatographic and MS conditions were selected for the sample measurements in this study. This method was used to perform the UHPLC-MS metabolic profiling analysis.

**Figure 5:**
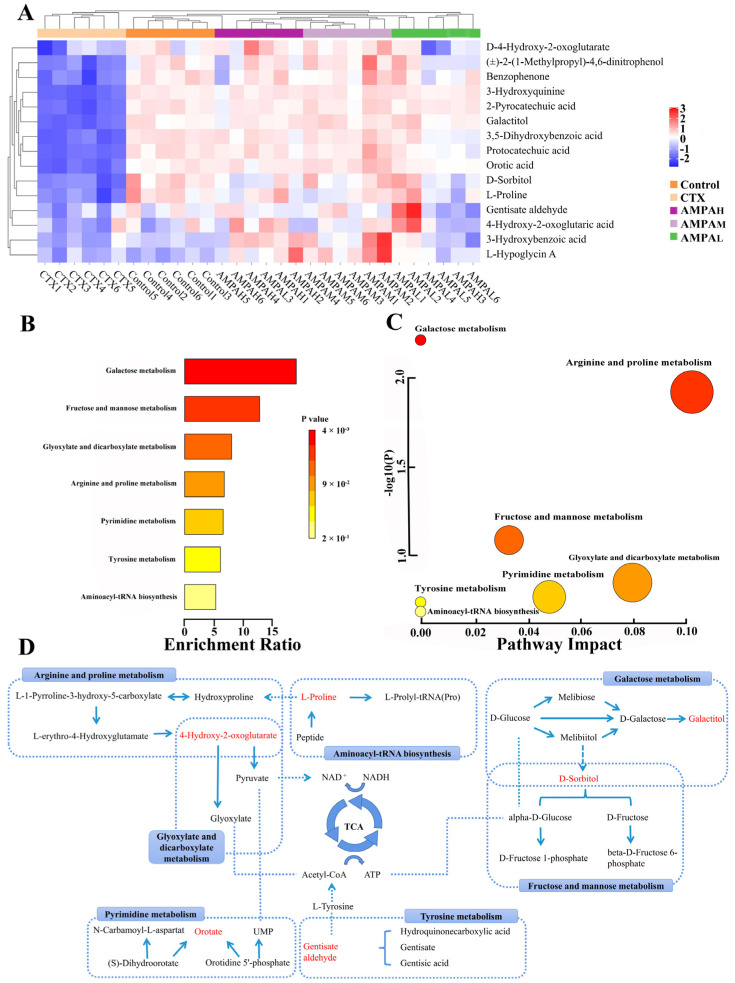
Heatmap showing the intensities of the potential biomarkers in each group (**A**), metabolic pathway enrichment analysis (**B**), metabolic pathway topology analysis (**C**), and the correlation networks between the main differential metabolites with the corresponding metabolic pathways (**D**).

The above results indicate that AMPA exerts a dose-dependent effect on the cyclophosphamide-induced immune regulation in immunodeficient mice. Furthermore, to better distinguish the difference between CTX and AMPA, subsequent experiments only analyzed the serum metabolism of the high-dose AMPA group. According to the OPLS-DA score plot, CTX and AMPA_H_ exhibited separation in the positive and negative models, demonstrating metabolite differences between the two groups. After further construction and differentiation by supervised orthogonal partial least squares discriminant analysis (OPLS-DA), different separation clusters could be observed in the OPLS-DA score map (OPLS-DA score plots). In order to prevent over-fitting of the OPLS-DA model, the quality was checked using the 200-response reciprocity test method. R2Y (cum) represents the cumulative explanatory rate of the model in the *y*-axis direction and Q2 (cum) represents the cumulative predictive power of the model. Generally, when the value of Q2 > 0.5, it indicates that the model is more stable and reliable. In this experiment, in the positive/negative ion modes, group AMPA_H_ vs. CTX obtained R2Y = 1, Q2 = 0.864 and R2Y = 0.998, Q2 = 0.989, respectively. The model quality was investigated using the OPLS-DA displacement test (OPLS-DA permutation test), as shown in Figure 4C,D, which proved that the OPLS-DA model was not overfitted in this experiment. The PCA score curves of the five groups showed an obvious separation trend, indicating that AMPA intervened in the serum metabolism of mice. Differential metabolites between groups were analyzed using multivariate statistical OPLS-DA. VIP > 1 and *p* < 0.05 were used as criteria for determining biologically significant differential metabolites in the OPLS-DA analysis, and then analysis carried out to visualize the with up-regulated expression (FC > 1) and with down-regulated expression (FC < 1). A univariate statistical RT analysis, accurate MS, and differential compounds were introduced into the metabolomics database HMDB (https://hmdb.ca/spectra/ms/search) (accessed 10 December 2022). Compared with the standard data information in the database, the quality error of all metabolites was less than 10 ppm; thus, the compounds were identified qualitatively. Fifteen potential biomarkers were identified and obtained (Table 1). The change trends in biomarkers in different groups are shown as a heatmap in Figure 5A. In contrast to the CTX group, the five differential metabolites of gentisate aldehyde, 4-hydroxy-2-oxoglutaric acid, D-sorbitol, galactitol, and L-proline displayed an increasing trend in immunoregulation, while the remaining ten metabolites showed a decreasing trend. The intervention with AMPA reversed these changes induced by CTX and produced a similar trend to that observed in the control group, indicating that AMPA exerted therapeutic and protective effects in the immunosuppressed mice.

### 2.8. Metabolic Pathway Analysis

To investigate the possible metabolic pathways by which AMPA protects CTX-induced immunodeficiency in mice, enrichment and topological analyses of 15 biomarker metabolic pathways were performed using the Metabolism option of MetaboAnalyst 5.0. (Table 2). The ordinate of the metabolite set enrichment overview diagram was the name of the metabolic pathway, and the horizontal coordinate was the enrichment ratio, as shown in Figure 5B,C. Each circle in the network topology analysis graph represents a metabolic pathway, and the change in the size and color of the circle represents the degree of influence of the metabolic pathway. The KEGG pathway enrichment analysis of the differential metabolites indicated that galactose metabolism, arginine and proline metabolism, tyrosine metabolism, fructose and mannose metabolism, glyoxylate and dicarboxylate metabolism, pyrimidine metabolism, and aminoacyl-tRNA biosynthesis occurred. These metabolic pathways are involved in the regulation of AMPA in glucose metabolism and immune activity in the body, and have a wide range of associations (Figure 5D). Galactose metabolism, tyrosine metabolism, and fructose and mannose metabolism metabolic pathways are mainly involved in the tricarboxylic acid cycle of polysaccharides, and tricarboxylic acid cycle metabolites are mainly involved in the activation of plant polysaccharides, immune cell activation, and immune factor expression, which have a wide range of immune regulatory functions [22,23]. The results of the present study are consistent with them.

## 3. Discussion

In this study, AMPA, an acid heteropolysaccharide, was tested for its immunomodulatory effects. Cell viability and NO production after the treatment of RAW 264.7 cells with AMPA were examined. The results suggest that AMPA significantly increased NO production but did not damage RAW 264.7 cells. The presence of NO in the macrophage media is considered to be one of the most reliable factors indicating the classical activation of macrophages [24] (Figure 1B). The increase in the expression levels of TNF-α, INF-γ, IL-2, and IL-6 in RAW264.7 cells suggest that AMPA can enhance immunoreactivity (Figure 1C–F). Immune cytokines, such as TNF-α, INF-γ, IL-2, and IL-6, have multiple functions in immunomodulation processes. In particular, cytokines released by immune cells can stimulate the innate immune response that is necessary for immunological regulation [21] (Figure 2D–I). The activation of the TLR4/MyD88/NF-κB signaling pathway mostly occurs during an immune response. It has been observed that TLR4 can interact with MyD88, thereby leading to the activation of NF-κB, which is indispensable for the production of cytokines, such as IL-2, IgM, and TNF-α. Therefore, AMPA promotes the secretion of immune factors TNF-α, INF-γ, IgM, IgA, IL-2, and IL-6, thereby activating the TLR4/MyD88/NF-κB signaling pathway, which could enhance immune function.

Furthermore, the immunomodulatory effects of AMPA in immunosuppressed mice were described and the effects that occur through the regulation of immune organs and cytokines were identified. As an effective immunosuppressive and chemotherapeutic agent, CTX can disrupt the DNA structure, interfere with the proliferation and differentiation of macrophages, kill immune cells, and weaken the body’s immune system [25]. CTX exhibits extensive toxicity; therefore, the CTX-induced immunosuppression model is most commonly used in the immune stimulation experiments [26]. The administration of CTX reduces the body weight, spleen index, and thymus index. These weights are considered as the key and intuitive indicators of non-specific immunity. The overall immune status of the body was assessed by assessing the thymus and spleen indices [27]. In this study, AMPA significantly increased the body weight, spleen index, and thymus index, indicating that AMPA could improve the immune function of the developing immune organs (Figure 2A–C). Moreover, immune factors were significantly increased, indicating that the AMPA treatment suppressed CTX-induced immunosuppression, which is an important limiting factor for the prognosis and recovery from chemotherapy in tumor patients. This suggests that CTX can enhance specific and non-specific host immunity, including both cellular and humoral immunities.

To further investigate the mechanism of the protective effect of AMPA on CTX-induced immunodeficiency, serum differential metabolites between the AMPA and CTX groups and the control and CTX groups were analyzed using UHPLC-MS. Through the tricarboxylic acid cycle in vivo, AMPA regulated metabolic pathways to promote the expression of immune factors and the generation of immune responses [28] (Figure 3). Recently, studies have revealed that tricarboxylic acid cycle metabolites play a wide range of roles in immune and inflammatory responses, both physiological and pathological. Most studies assessing immune cells, immune factors, and glycometabolites have revealed some clear correlations [29]. As it is well known, the TCA cycle is an important hub for the metabolism of three major substances, i.e., carbohydrates, fats, and proteins, and provides an energy source for mammals [30,31]. Lactate dehydrogenase proton-linked monocarboxylate catalyzes a primary TCA reaction with through the oxidation of lactate to pyruvate. For the faster recovery of ATP, glucose glycolysis in vivo can produce energy quicker, as glucose can also serve as a transport carrier of enzymes in the body, promoting the TCA cycle and the flow of energy and material [32], and regulating galactose metabolism and fructose and mannose metabolism. Lactose alcohol and sorbitol produced by galactose metabolism are not absorbed and digested in the small intestine, but are fermented and decomposed by microorganisms in the large intestine. They exhibit complex molecular energy transfer in the human body, which are upregulated by the metabolites of lactose alcohol and sorbitol, maintaining a stable state of the metabolic pathway in the organism and preventing diseases [33]. Sorbitol is metabolized to glucose by glycolysis, and red blood cells absorb fructose and mannose in the presence of glucose, which determine the metabolic status and maintain ATP production through lactate, proline, and glucose [34]. Proline participates in metabolic pathways, including nitric oxide and proline, as well as in the expression of NO and iNOS in macrophages and plays an immunoprotective role during activation [35]. The downregulation of D-4-hydroxy-2-oxoglutarate in the arginine and proline metabolic pathway produces the secondary metabolites pyruvate and glyoxylate, producing NAD+, and NAD+ coenzymes maintain the activity of numerous metabolic and non-metabolic enzymes. Meanwhile, mitochondria are responsible for the conversion of pyruvate and glyoxylate to acetyl-CoA, stimulating glycolysis [36] and promoting the glycolytic pathway, which plays a key role in enhancing immune factor expression. Lactate nucleoside achieves lactate clearance and UMP by nucleotide reaction; UMPA is considered to be one of the hub molecules in pyrimidine metabolism and plays a certain immunomodulatory role [37]. These experimental results provide a useful basis for developing AMPA for immune protective effects and demonstrate that Armillaria mellea could be developed as a potential natural product for enhancing immunity to prevent immune-related diseases.

## 4. Materials and Methods

### 4.1. Preparation of AMPA

Armillaria fruiting bodies were obtained by hot water extraction and ethanol precipitation to obtain Armillaria polysaccharides. Then, the samples were purified by a DEAE cellulose column (3 cm and 75 cm); purified with 1 mol/L sodium chloride at a titration rate of 1 mL/min; and dialyzed, desalted, and lyophilized to obtain crude samples. The crude product was purified using a Sephadex G-6b column (3 cm and 60 cm) and eluted with 1 mL/min of 1 mol/L sodium chloride. The final samples were an acid polysaccharide obtained after concentration and lyophilization. This mainly contained mannose (Man), glucuronic acid (GlcA), glucose (Glc), galactose (Gal), arabinose (Ara), and fucinose (Fuc) with a molar ratio of 0.18:0.06:0.21:0.15:0.05:0.02. The molecular weight was 7.323 × 103 Da.

### 4.2. Materials and Instruments

Mouse macrophage cells (RAW264.7 cells) were purchased from Changsha Yingrun Biotechnology Co., Ltd. (Changsha, China). Enzyme-linked immunosorbent assay kits of tumor necrosis factor (TNF)-α, interleukin (IL)-2, interleukin (IL)-6, immunoglobulin A (Ig A), immunoglobulin M (IgM), and interferon (INF)-γ were purchased from Hushi Kenke Biotechnology Co., Ltd. (Huangshi, China). The other chemicals were of analytical grade.

### 4.3. RAW264.7 Cells for the Experiments

#### 4.3.1. Evaluating the Optimal Concentration of AMPA and RAW264.7

Murine macrophage cells (RAW264.7) were pre-cultured in DMEM (Gibco, Shanghai, China) and augmented with 10% heat-incapacitated fetal bovine serum (FBS, BI, Herzliya, Israel), 100 U/mL of streptomycin, and 100U/mL of penicillin G (HyClone, Beijing, China) in an incubator at 37 °C with 5% CO_2_ until the cells reached 70% confluency.

The cells were plated in 96-well cell culture plates with 6 complex wells for each group to adjust the cell density to 2 × 10^5^/mL. Concentration values of 25, 50, 100, 200, and 400 μmol/L were used for AMPA and 1 μmol/L for LPS. After 24 h of incubation, the supernatant was discarded and different concentrations of the acid polysaccharide were added. After incubation for 24h in a 37 °C, 5% CO_2_ incubator, 10 μL CCK8 per well were exposed to the light in a 37 °C, 5% CO_2_ incubator for 4 h before measuring the absorbance (OD) at 450 nm.

#### 4.3.2. Assessment of NO Production and ELISA Kits

Log-growth-phase RAW264.7 cells were seeded at a rate of 2.0 × 10^5^ cells/well in 6-well plates for 24 h. The experiment was divided into control, LPS, and AMPA group (25 μg/mL, 50 μg/mL, and 100 μg/mL). The cell culture supernatant was collected and centrifuged at 3000 rpm for 20 min. One sample was used to determine the effect of AMPA on NO production and the other was used to detect the cell abundance of IL-6, IL-2, IgA, IgM, TNF-α, and IFN-γ using the corresponding ELISA kit.

#### 4.3.3. Western Blot Analysis of the AMPA-Activated RAW264.7 Cells

The detection of protein expression was performed using the Western blot technique. Briefly, proteins were extracted with NP-40 lysis buffer containing phosphatase inhibitors, separated by 10% SDS-PAGE electrophoresis, and transferred to polyvinylidene fluoride (PVDF) membranes. Based on the protein expression, the PVDF membranes were incubated with anti-β-actin/anti-TLR4/anti-P65/anti-P65/anti-MyD88 (Boster Biological, Wuhan, China) and left overnight at 4 °C. After washing with TBST, the PVDF membranes were incubated with horseradish-peroxidase-conjugated secondary antibodies (Boster Biological, Wuhan, China) for 2 h at room temperature. After washing with TBST, the protein bands on the PVDF membrane were detected using NcmECL Ultra (NCM Biotechnology, Suzhou, China).

### 4.4. Animals and Treatment

Sixty KM mice (male; SPF grade; 10–22 g) were purchased from the Experimental Animal Center of Jilin University (permission code SCXK 2021-0002, Jilin, China). The mice were kept in a constant environment (12 h light/dark cycle; 25 °C ± 2 °C temperature; 55% ± 5% relative humidity) and housed in separate cages (twelve mice per cage). After 7 days of adaptive feeding, they were randomly divided into 5 groups: control group (0.9% saline), CTX group (0.9% saline), and high, medium, and low doses of AMPA (50, 100, and 200mg/kg·d, respectively). These doses were calculated based on the glucose content, which were 10, 20, and 30 times greater than the daily recommended human dose, respectively. Except for the blank group, the remaining group received cyclophosphamide 80 mg/kg·d from the beginning to days 24, 25, and 26 to establish the immunodeficiency model. After the administration, each batch of mice was subjected to subsequent index detection and investigation.

After 30 days of continuous intervention, mice were fasted overnight for 12 h; eyeball blood was collected, and the plasma was kept at room temperature for 60 min and then centrifuged at 3000 rpm and 4 °C for 15 min, frozen in liquid nitrogen, and stored in a −80 °C refrigerator. Before analysis, the serum samples were removed from the −80 °C refrigerator and thawed in a 4 °C refrigerator, and a portion of mouse serum was used to determine the biochemical indexes, including IL-2, IL-6, IgM, IgA, TNF-α, and IFN-γ content. The other part was used for the UHPLC-MS analysis: A total of 100 μL of serum was collected, 300 μL of methanol (3 × methanol, 4 °C) was added, vortexing was performed for 30 s, and then centrifugation at 12,000 rpm, 4 °C for 15 min. The supernatant was removed, nitrogen-dried, reconstituted with 1 mL ultrapure water (4 °C), vortexed for 15 s, centrifuged again at 12,000 rpm and 4 °C for 15 min, then filtered through a 0.22 μm filter membrane, and finally placed in an injection vial for UHPLC-MS/MS detection.

The weighted spleen samples (40 mg) were cut to pieces and placed in a 2 mL tube. The sample was mixed with 200 μL N-40 reagent and a stainless steel bead. The proteins were extracted using NP-40 reagent with a tissue homogenizer at 4 °C. After the determination of the protein concentration using the BCA assay kit, the spleen proteins were subjected to Western blot analysis.

### 4.5. Serum UPLC-MS Metabolomic Analysis

Chromatographic conditions: A Thermo Golden C18 column (50 × 2.1 mm, 1.9 μm) was used. The column temperature box was set as 35 °C. The sample load plate temperature was 4 °C. The mobile phase A was 0.1% formic acid–water, and the phase B was 0.1% formic acid–chromatographic acetonitrile. The flow rate was 0.3 mL/min- The gradient elution was 0–1 min, 3% B, 1–16 min, 3–68% B, 16–18 min, 68% B, 18–25 min, 68–90% B, 25–26 min, 90–100% B, 26–32 min, 100% B. The injection volume was 2.0 μL.

Mass spectrum conditions: An electrospray ion source (ESI) was used. The mass spectrum parameters were 6.125 Mpa; the auxiliary gas flow rate was 2.625 Mpa; the purge air flow was 0.175 Mpa; the spray voltage was 3.5 kV; the capillary temperature was 320 °C; the auxiliary gas and heat source temperature was 350 °C; the scanning mode was Full MS/dd-MS2 (TopN = 5) positive and negative ion scanning mode, Full MS, 17, 500 (data-dependent MS^2^). In the MS/MS mode, the gradient collision energy (NCE) was 10%, 20%, and 40%; data were recorded and analyzed using Xcalibur software (v 2.2.42, Thermo Fisher Scientific, San Jose, CA, USA).

Serum samples were randomly analyzed by UHPLC-ESI-Q-Exactive Orbitrap/MS, and QC samples were used to ensure the stability of the system. QC samples were prepared by mixing small portions of the same volume of all serum samples to be tested. Before the sample analysis, the six QC samples were run to balance the system; blank samples (50% acetonitrile–water) and one QC sample were interspersed to test the stability of the system. After all samples, the column was eluted with a pure water–methanol solution and finally stored in pure methanol.

### 4.6. Statistical Analysis

The raw data of the mass spectrometry were introduced into the software for retention time (t_R_) correction, peak identification, peak extraction, and peak integration. The resulting data matrix was normalized and imported into the SIMCA 14.1 software for multivariate statistical analysis. According to the orthogonal partial least squares discriminant analysis (OPLS-DA) model, the variable projection importance value (VIP > 1) and the independent sample t-test (*p* < 0.05) screened the differential metabolites through HMDB (http://www.hmdb.ca/) (accessed 10 December 2022), and the screened differential metabolites were retrieved and identified. An enrichment analysis of the differential metabolites was performed using the KEGG (https://www.kegg.jp/) (accessed 10 December 2022)database. The metabolic pathway analysis was performed using MetaboAnalyst 5.0 (https://www.metaboanalyst.ca/) (accessed 10 December 2022). For the statistical analysis of the data, the SPSS 27.0 software was used, and the measurement data are expressed as x ± s (with a one-way ANOVA for comparison between multiple groups; for the *t*-test, *p* < 0.05 and *p* < 0.01 were considered statistically significant).

## 5. Conclusions

In the present study, untargeted serum metabolomics combined with immune cell activity was successfully applied to investigate the immune-protective effects of AMPA. The results show that, in vitro, compared with LPS-induced RAW264.7 cells, the AMPA treatment elevated the levels of the cellular immune factors IL-2, IL-6, IgM, IgA, TNF-α, and IFN-γ; promoted the expression of immune proteins; and activated the TLR4/MyD88/NF-κB signaling pathway to produce immunological responses. Cellular immune factor secretion and signaling pathway activation were also demonstrated in the spleens of cyclophosphamide-induced immunosuppressed mice model in vivo. A univariate statistical analysis based on UHPLC-MS was used to screen the metabolites with difffferent contents in the sera of mice after 30 days of intervention. Combined with the enrichment analysis of the KEGG metabolic pathway, six different metabolites produced by AMPA body’s glucose metabolism and the seven metabolic pathways involved were screened. The metabolic pathways involved in regulation include six key differential metabolites, which mainly regulate three metabolic pathways involved in the tricarboxylic acid cycle of polysaccharides: galactose metabolism, tyrosine metabolism, and fructose and mannose metabolism. These represent a potential target for AMPA to regulate immune activity. The acid polysaccharide of Armillaria mellea and its main immunomodulatory mechanism were studied. The results of this study provide new insights into the role of serum metabolites in the regulatory mechanisms of immune activity, facilitating the understanding of the immunoprotective activity of AMPA. This study lays a theoretical foundation for the further development and utilization of *Armillaria mellea* dietary supplements to improve immunity, as well as for the subsequent development of AMPA-related products.

## Figures and Tables

**Table 1:** Statistical analysis results of the potential biomarkers in the serum.

ID	Rt (min)	Query Mass	Metabolite Name	Formula	Ion Forms	Compound ID	KEGG	VIP	FC		Change Tend	
									CTX/ Control	AMPA/ CTX	CTX/ Control	AMPA/ CTX
1	6.8	90.5268	D-4-Hydroxy -2-oxoglutarate	C_5_H_6_O_6_	M+H+NH_4_	HMDB 0060466	C05946	1.21	0.95	0.86	↓	↓
2	9.7	90.5268	Gentisate aldehyde	C_7_H_6_O_3_	M+2H	HMDB 0004062	C05585	1.14	0.96	1.13	↓	↑
3	11.2	90.5268	3-Hydroxybenzoic acid	C_7_H_6_O_3_	M+2H	HMDB 0002466	C00587	1.33	0.95	0.96	↓	↓
4	11.4	205.0609	(±)-2-(1-Methylpropyl)-4,6-dinitrophenol	C_10_H_12_N_2_O_5_	M+H-2H_2_O	HMDB 0032559	C14302	1.21	0.98	0.97	↓	↓
5	13.4	205.0609	Benzophenone	C_13_H_10_O	M+Na	HMDB 0032049	C06354	1.02	0.99	0.97	↓	↓
6	16.9	90.5268	Hydroxy -2-oxoglutaric acid	C_5_H_6_O_6_	M+H+NH_4_	HMDB 0002070	C01127	1.09	0.96	1.09	↓	↑
7	18.4	90.5267	L-Hypoglycin A	C_7_H_11_NO_2_	M+H+K	HMDB 0029427	C08287	1.21	0.96	0.98	↓	↓
8	29.8	166.0982	3-Hydroxyquinine	C_20_H_24_N_2_O_3_	M+H-H_2_O	HMDB 0001091	C07344	1.08	0.96	0.96	↓	↓
9	8.6	177.0077	3,5-Dihydroxybenzoic acid	C_7_H_6_O_4_	M+Na	HMDB 0013677	C00180	1.17	0.98	0.95	↓	↓
10	20.5	205.0609	D-Sorbitol	C_6_H_14_O_6_	M+Na	HMDB 0000247	C00794	1.34	0.97	1.34	↓	↑
11	10.3	177.0077	Protocatechuic acid	C_7_H_6_O_4_	M+Na	HMDB 0001856	C00230	1.14	0.97	0.95	↓	↓
12	13.1	177.0078	Galactitol	C_6_H_14_O_6_	M+Na	HMDB 0000107	C01697	1.23	0.96	1.23	↓	↑
13	18.9	255.9450	2-Pyrocatechuic acid	C_7_H_6_O_4_	M+Na	HMDB 0000397	C00196	1.19	0.97	0.94	↓	↓
14	21.8	205.0607	L-Proline	C_5_H_9_NO_2_	M+H-2H_2_O	HMDB 0000162	C00148	1.10	0.98	1.10	↓	↑
15	12.0	177.0076	Orotic acid	C_5_H_4_N_2_O_4_	M+H-H_2_O	HMD B0000226	C00295	1.24	1.23	0.94	↑	↓

**Table 2:** Pathway analysis of biomarkers using MetaboAnalyst 5.0 online.

Pathway Name	Match Status	*p*	−log(*p*)	Holm *p*	FDR	Impact
Galactose metabolism	2/27	0.0061524	2.211	0.5168	0.50498	0.0
Arginine and proline metabolism	2/38	0.012023	1.92	0.99793	0.50498	0.10165
Fructose and mannose metabolism	1/18	0.08088	1.0922	1.0	1.0	0.03313
Glyoxylate and dicarboxylate metabolism	1/32	0.13985	0.85435	1.0	1.0	0.07937
Pyrimidine metabolism	1/39	0.16809	0.77445	1.0	1.0	0.04819
Tyrosine metabolism	1/42	0.17995	0.74484	1.0	1.0	0.0
Aminoacyl-tRNA biosynthesis	1/48	0.20324	0.69199	1.0	1.0	0.0

## Data Availability

Data are contained within the article.
